# Analysis of Compressive Strength of Anhydrite Binder Using Full Factorial Design

**DOI:** 10.3390/ma16186265

**Published:** 2023-09-18

**Authors:** Dalia Nizevičienė, Nora Kybartienė, Vacius Jusas

**Affiliations:** 1 Faculty of Electrical and Electronics Engineering, Kaunas University of Technology, Studentų Str. 48, LT-50254 Kaunas, Lithuania; dalia.nizeviciene@ktu.lt; 2 Faculty of Chemical Technology, Kaunas University of Technology, Radvilėnų Str. 19, LT-50254 Kaunas, Lithuania; nora.kybartiene@ktu.lt; 3 Faculty of Informatics, Kaunas University of Technology, Studentų Str. 50, LT-50254 Kaunas, Lithuania

**Keywords:** full factorial design, FGD gypsum, anhydrite binder, compressive strength, calcination temperature, activators

## Abstract

Flue gas desulfurization gypsum (FGD gypsum) is obtained from the desulphurization of combustion gases in fossil fuel power plants. FGD gypsum can be used to produce anhydrite binder. This research is devoted to the investigation of the influence of the calcination temperature of FGD gypsum, the activators K_2_SO_4_ and Na_2_SO_4_, and their amount on the compressive strength of anhydrite binder during hydration. The obtained results showed that as the calcination temperature increased, the compressive strength of anhydrite binder decreased at its early age (up to 3 days) and increased after 28 days. The compressive strength of the anhydrite binder produced at 800 °C and 500 °C differed more than five times after 28 days. The activators K_2_SO_4_ and Na_2_SO_4_ had a large effect on the hydration of anhydrite binder at its early age (up to 3 days) in comparison with the anhydrite binder without activators. The presence of the activators of either K_2_SO_4_ or K_2_SO_4_ almost had no influence on the compressive strength after 28 days. To determine which factor, the calcination temperature of FGD gypsum (500–800 °C), the hydration time (3–28 days) or the amount (0–2%) of the activators K_2_SO_4_ and Na_2_SO_4_, has the greatest influence on the compressive strength, a 2^3^ full factorial design was applied. Multiple linear regression was used to develop a mathematical model and predict the compressive strength of the anhydrite binder. The statistical analysis showed that the hydration time had the strongest impact on the compressive strength of the anhydrite binder using activators K_2_SO_4_ and Na_2_SO_4_. The activator K_2_SO_4_ had a greater influence on the compressive strength than the activator Na_2_SO_4_. The obtained mathematical model can be used to forecast the compressive strength of the anhydrite binder produced from FGD gypsum if the considered factors are within the same limiting values as in the suggested model since the coefficient of determination (R2) was close to 1, and the mean absolute percentage error (MAPE) was less than 10%.

## 1. Introduction

Synthetic gypsum is produced as a by-product of an industrial process. Flue gas desulfurization (FGD) gypsum is one of the varieties of synthetic gypsum. In 2020, global FGD gypsum production reached 255 million tons [1]. Most of the FGD gypsum was produced in Asia (55%), followed by Europe (22%), North America (18%) and the rest of the world (5%) [1]. FGD gypsum is obtained from the desulphurization of combustion gases in fossil fuel power plants. The combustion of sulphureous fossil fuels, such as anthracite, bituminous coal, lignite and oil, produces sulphur dioxide (SO_2_), which, if it is not removed in a flue gas desulphurization plant, escapes into the atmosphere with the flue gases [2]. Sulphur dioxide is one of the causes of atmospheric and environmental pollution. Acid rain results when sulphur dioxide is emitted into the atmosphere [3]. Acid rain has a negative impact on soil, aquatic ecosystems, forest trees, crop plants, buildings, physiological activities of animals and human health [4,5].

The most popular method to remove sulphur dioxide from flue gas is the wet scrubbing method. The flue gas is countercurrent washed with aqueous suspensions of limestone (CaCO_3_) or lime (Ca(OH)_2_), producing calcium sulphite (CaSO_3_) in aqueous suspension. Finally, the calcium sulphite is further oxidized with atmospheric oxygen to calcium sulphate dihydrate (CaSO_4_∙2H_2_O) or gypsum, which is called flue gas desulfurization gypsum (FGD gypsum) [2]. Usually, FGD gypsum has a higher purity (96–99%) [6] and lower level of impurities than most natural gypsum (80–96%) [7]. Because FGD gypsum is mainly composed of calcium sulphate dihydrate (CaSO_4_·2H_2_O), it can replace natural gypsum without further processing [8]. Therefore, using FGD gypsum, being a by-product, can reduce the environmental effect of the process.

FGD gypsum can be a product that has many applications in different industries. It is used in agriculture [9,10], civil engineering [11,12], water treatment [13], sorption of phosphorus ions [14] and the glass industry [2]. FGD gypsum is widely applied in the construction industry as a setting retarder in Portland cement [8,15,16,17], for producing calcium sulphoaluminate cement [18], as high-strength building materials [19] and as a component of gypsum plaster [20]. By adding the quaternary phase (Q phase, Ca_20_Al_13_Si_3_Mg_3_O_68_) or cement and mineral powder to FGD gypsum, it is possible to reduce the water solubility of FGD gypsum and the water absorption, increase the strength, accelerate the hydration reaction and improve microstructure [21,22].

FGD gypsum can be used to produce α-calcium sulphate hemihydrate (α-CaSO_4_∙0.5H_2_O), β-calcium sulphate hemihydrate (β-CaSO_4_∙0.5H_2_O) or calcium sulphate (anhydrite, CaSO_4_). The manufacturing and utilization of α-CaSO_4_∙0.5H_2_O were widely investigated in [23,24,25,26]. Miao et al. [23] synthesized calcium sulfate hemihydrate whiskers from raw FGD gypsum via phase transition in a CaCl_2_ solution under atmospheric pressure. Liu et al. [24] prepared calcium sulfate whiskers from FGD gypsum via hydrothermal synthesis using additives of magnesium chloride, citric acid and sodium dodecyl benzene sulfonate. Yang et al. [25] obtained alpha-calcium sulfate hemihydrate from FGD gypsum using the salt solution method under atmospheric pressure. Li et al. [26] successfully made fire-resistant panels from pure FGD gypsum, pure commercial gypsum and different FGD gypsum/fly ash and pure commercial gypsum/fly ash mixtures. The FGD gypsum/fly ash panels presented a relatively lower density, lower water absorption and lower mechanical strength but better insulating capacities than the commercial gypsum/fly ash panels.

However, few works were focused on β-CaSO_4_∙0.5H_2_O [27] and CaSO_4_ production [28,29,30,31]. Maiti et al. [27] prepared lightweight plasters using FGD gypsum and exfoliated vermiculite, which can be used for interior wall applications. Calcination of FGD gypsum at a temperature range of 500–750 °C and the use of soluble activators enabled the production of a high-performance anhydrite binder [28,29]. The FGD gypsum calcined at 650 °C for 4 h was used in the mixture of Portland cement to produce self-levelling floors [30]. Anhydrite cement was obtained from FGD gypsum, which was burnt together with activators, such as glass and cupola dust, at 800 and 900 °C [31].

In the reviewed works [27,28,29,30,31], the influence of the calcination temperature and additives on the properties of anhydrite was investigated, but it was not determined which factor had the greatest influence on compressive strength. Therefore, the aim of this research was to investigate the impact of the factors of the calcination temperature of FGD gypsum (500–800 °C), the hydration time (3–28 days) and the amount (0–2%) of activators (K_2_SO_4_ and Na_2_SO_4_) on the compressive strength of an anhydrite binder produced from FGD gypsum using a 2^3^ full factorial (DOE) design and to develop a mathematical model using multiple linear regression to predict the compressive strength of the anhydrite binder within the defined limits.

## 2. Materials and Methods

### 2.1. Materials

In this study, FGD gypsum powder was taken from the storage tank of a fuel-burning plant in Lithuania, dried at 40 ± 2 °C and sieved through a 0.5 mm sieve.

The results of chemical composition (Table 1) showed that FGD gypsum was of high purity, and CaSO_4_∙2H_2_O was 96.22%. The pH was 6.45. The specific surface area *S_spec_* was less than 100 m^2^/kg.

**Figure 1:**
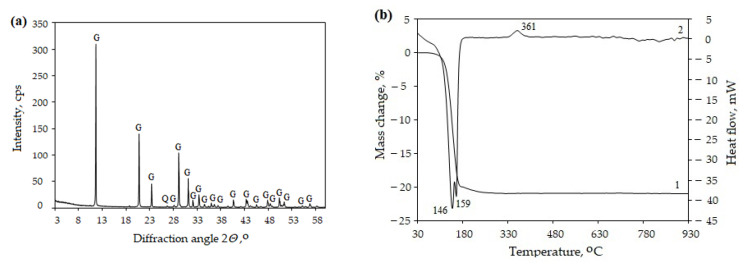
XRD pattern (**a**) and STA curves (**b**) (1—TG, 2—DSC) of FGD gypsum. Indexes: G—gypsum (PDF No. 00-033-0311), Q—quartz (PDF No. 00-046-1045).

The X-ray diffraction (XRD) analysis (Figure 1a) confirmed that gypsum CaSO_4_∙2H_2_O dominates in FGD gypsum. The results of simultaneous thermal analysis (STA) (Figure 1b) showed that gypsum dehydrated, and water evaporated in temperature range of 105–190 °C. Insoluble anhydrite formed at temperatures between 340 °C to 370 °C.

K_2_SO_4_ and Na_2_SO_4_ were used as activators to accelerate the hydration and hardening of anhydrite binder. Either 1% or 2% of activator by the weight of anhydrite was added together with the gauging water. Since unground anhydrite binder was used, water-soluble sulphate activators were selected for this research. Another advantage of these activators is that they require a small amount (≤2%) for hydration activation.

### 2.2. Methods

X-ray fluorescence spectroscopy (XRF) was carried out with a Bruker X-ray S8 Tiger WD (Bruker AXS, Karlsruhe, Germany) spectrometer equipped with a Rh tube with an energy level of up to 60 kV. The powder was passed through a 0.08 mm sieve and pressed into 5 × 40 mm cylindrical tablets. The samples were measured in He atmosphere. SPECTRAplus QUANT EXPRESS software v2.0 was used to analyse the data [32].

X-ray diffraction analysis (XRD) was performed on a D8 Advance diffractometer (Bruker AXS, Karlsruhe, Germany) with Bragg–Brentano geometry using Ni-filtered CuKα radiation and graphite monochromator operating at 40 kV and 45 mA. The measurement range was 3–70° (2*θ*) in steps of 2*θ* = 0.02°. The samples were passed through a 0.08 mm sieve. The X-ray diffraction patterns were identified using references available in PDF-2 database [33].

Simultaneous thermal analysis (STA) was carried out on a LINSEIS STA PT 1000 (Linseis Messgeräte GmbH, Selb, Germany) thermal analyser with ceramic sample handlers and crucibles of Pt-Rh. The heating rate was set to 10 °C/min, and the range of temperature was from 25 °C up to 945 °C under N atmosphere. The powder was passed through a 0.08 mm sieve.

Scanning electron microscopy (SEM) was carried out using a LEO 440 SEM (SGC Equipment, Austin, TX, USA) equipped with an EDS system OXFORD ISIS Link and Si (Li) PENTAFET detector (Oxford Instruments, Abingdon, UK) using an accelerating voltage of 20 kV at working distance of 10 mm.

The chemically combined water (Ignition loss, %) of FGD gypsum and of hydrated anhydrite binder was calculated after heating the material at 400 °C. The hydration of samples was stopped with isopropyl alcohol and was dried at 40 ± 2 °C. The degree of hydration was calculated by measuring the weight loss in the hydrated samples.

FGD gypsum was passed through a 0.5 mm sieve and calcined at temperature range of 500–900 °C for 1 h in a muffle furnace. As the result, an anhydrite binder was obtained.

Density was measured with automatic gas pycnometer Quantachrome Instruments Ultrapyc 1200e (Profcontrol GmbH, Potsdam, Germany). The Blaine method was used to determine the specific surface area *S_spec_* in accordance with the European Standard EN 196-6 [34].

The 2 × 2 × 2 cm cubic samples were formed from anhydrite binder paste of normal consistency to find out the mechanical characteristics of the anhydrite binder. The water/anhydrite binder ratio and the setting time of the mixture were determined according to the standard EN 196-3 [35]. Samples were hardened in 90% relative humidity environment for 28 days and compressed by press ELE AutoTest (ELE International, Milton Keynes, UK). The compressive strength of anhydrite binder samples was measured according to the standard EN 196-1 [36]. In addition, after 28 days of hydration, the samples were dried at 50 ± 2 °C and compressed.

Full factorial (DOE) design for three independent factors at two levels (2^3^) was used to study the effect of factors on a response. Calcination temperature of FGD gypsum, hydration time and amount of activators were chosen as independent factors. Compressive strength of anhydrite binder was chosen as a response. Each factor had two levels, low and high, coded as (−1) and (+1). The specific dosage of the considered factors is provided in Section 3.3. Eight experimental runs were performed for three independent factors [37].

Multiple linear regression as the statistical method was used in DOE data analysis [38]. It shows the relationship between multiple independent factors and a response. A general multiple linear regression model is presented by the following formula [37,39]:

(1) $$Y=b_{0}+\sum_{i=1}^{k}b_{i}x_{i}+\sum_{\begin{array}{c} i,j=1\\ i<j \end{array}}^{k}b_{ij}x_{i}x_{j}$$

where:

*Y*—a response;
*x_i_*, *x_j_*—an independent factor;
*b*_0_—an intercept;
*b_i_*—a linear coefficient;
*b_ij_*—an interaction coefficient.

The Student‘s *t*-test was used to evaluate the significance of coefficients in multiple regression. The calculations were performed at 95% confidence interval and a significance level of *p* less than 0.05 [40]. The sign of coefficient indicates whether the response increases (+) or decreases (−) as independent factor increases. The value of coefficient shows how much the mean of response changes when one independent factor increases by one unit while other factors are held constant [37]. Fisher’s test was used to evaluate the adequacy of model [40]. The calculations were performed at 95% confidence interval and a significance level of *p* less than 0.05 [40]. The quality (suitability) of the multiple linear regression model fitness was expressed with the coefficients of determination (*R*^2^) and the mean absolute percentage error (MAPE). The model is a good fit for the data if *R*^2^ value is in the range of 0.8–1. The model is highly accurate if MAPE is less than 10% [41].

## 3. Results and Discussion

### 3.1. Calcination Temperature

**Figure 2:**
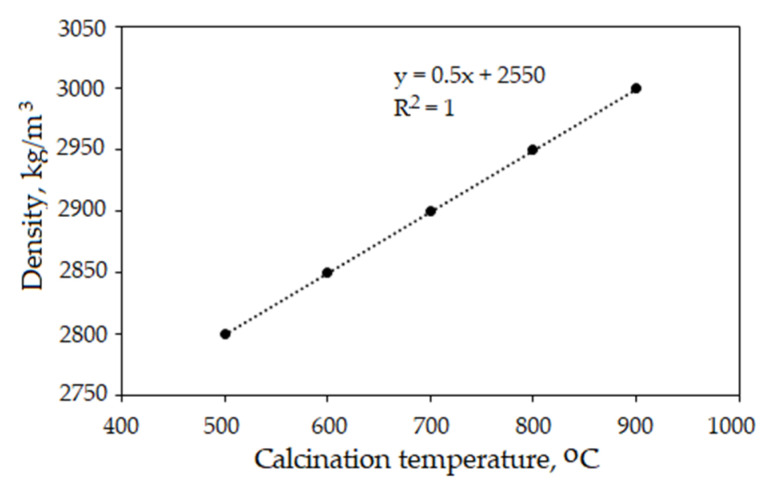
Effect of calcination temperature of anhydrite on density.

The FGD gypsum was calcined in a temperature range of 500–900 °C. The physical and mechanical properties of the anhydrite binder were investigated. The size and surface of the anhydrite particles have an influence on the properties of the anhydrite. To determine the impact of the calcination temperature on the particles of the anhydrite, the density was measured, and SEM was performed. The results showed that the density of the anhydrite calcined at different temperatures increased gradually (Figure 2). The relationship between the density and the calcination temperature can be described with a linear regression function. The coefficient of determination *R*^2^ indicated a perfect correlation (*R*^2^ = 1).

**Figure 3:**
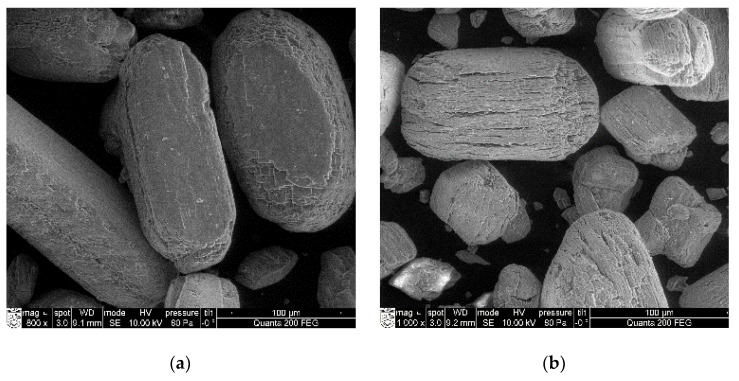

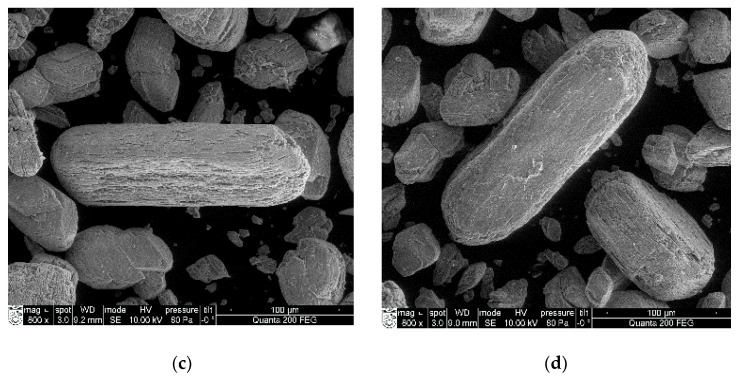
SEM of FGD gypsum (**a**) and anhydrite produced at 500 °C (**b**), 700 °C (**c**) and 900 °C (**d**).

SEM analysis (Figure 3) confirmed that an increased calcination temperature enabled the formation of the denser particles. Similar results were obtained by Elert et al. [42].

**Figure 4:**
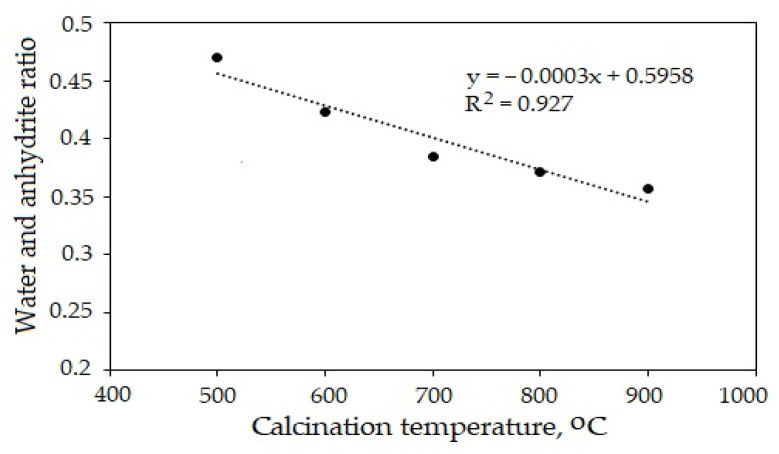
Effect of calcination temperature of anhydrite on water and anhydrite ratio.

In order to determine the effect of the calcination temperature on the mechanical properties, the water (W) and anhydrite (A) ratio (W/A) were determined. The results showed that the water and anhydrite ratio (W/A) decreased from 0.47 to 0.35 as the calcination temperature increased (Figure 4). The Pearson’s correlation coefficient *r* (−0.96) indicated a very high negative linear relationship between the W/A and the calcination temperature. The coefficient of determination *R*^2^ showed a very high correlation (*R*^2^ = 0.927).

The cubic (2 × 2 × 2 cm) samples were formed for each calcination temperature (500, 600, 700, 800 and 900 °C). The samples were hardened in a 90% relative humidity environment at 23 ± 2 °C. The compressive strength was determined after 3 and 28 days of curing.

As the calcination temperature increased, the compressive strength of the anhydrite binder decreased at its early age (up to 3 days) and increased after 28 days (Table 2). After 28 days, the compressive strength of the anhydrite binder produced at 900 °C and 500 °C differed more than six times.

In summary, as the calcination temperature of FGD gypsum increased, the anhydrite particles became smaller and denser, a smaller amount of gauging water was needed for the anhydrite binder and the anhydrite binder became stronger. The obtained results were similar to the results of Ludwig et al. [28].

### 3.2. Activators and Amount of Activator

The influence of the activators (K_2_SO_4_ and Na_2_SO_4_) and the amount of activator (1% or 2%) on the hydration of the anhydrite binder produced at 500 °C and 800 °C was examined. The degree of hydration at different times of hydration was calculated, and XRD analysis was performed.

**Figure 5:**
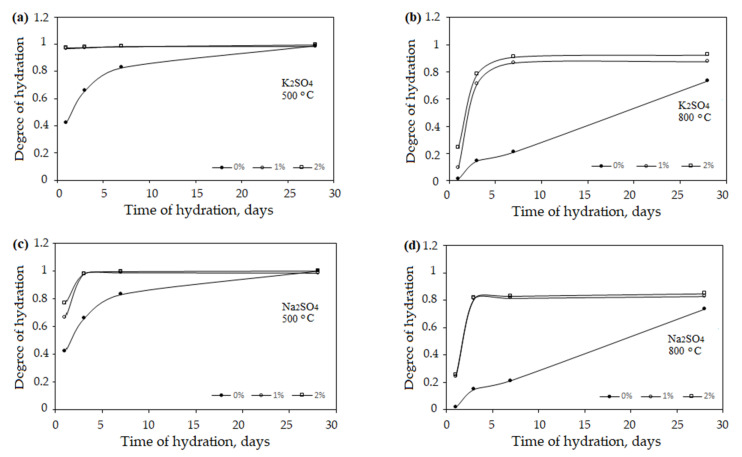
Degree of hydration of anhydrite produced at 500 °C and 800 °C temperature using K_2_SO_4_ (**a**,**b**) and Na_2_SO_4_ (**c**,**d**).

The obtained results showed that anhydrite binder obtained at 500 °C (Figure 5a) hydrates faster than anhydrite binder obtained at 800 °C (Figure 5b).

The anhydrite binder obtained at the temperature of 500 °C was fully hydrated after 28 days (degree of hydration was 0.99), while the degree of hydration of the anhydrite binder obtained at the temperature of 800 °C was only 0.73. The hydration of the anhydrite binder was influenced by the structure of the material. At the temperature of 500 °C, the water dispersed the gypsum crystals. Their surface was crannied (Figure 3b). Such a material had a higher internal porosity and needed more gauging water (W/A = 0.47, Figure 4). The anhydrite bound and hardened very quickly (initial setting time was 2 min, and the final setting time was 3 min), but the gypsum stone was not very strong (compression strength after 3 days was 2.04 MPa, Table 2). At the temperature of 800 °C, the crystal lattice of the material became denser, and less water was needed (W/A = 0.37, Figure 4). The anhydrite set and hardened slowly (initial setting time was 100 min, and the final setting time was 135 min), and the compressive strength after 3 days of hydration was 0.51 MPa, Table 2). The activators were needed to accelerate hydration.

When using both K_2_SO_4_ (Figure 5a,b) and Na_2_SO_4_ (Figure 5c,d) as activators, the hydration was significantly activated. After burning the FGD gypsum at a temperature of 500 °C and using the activator K_2_SO_4_, the degree of hydration already reached 0.95 (Figure 5a) after 1 day, which was more than two times faster than without using the additive (the degree of hydration was 0.42). When using the additive Na_2_SO_4_, the hydration was slower (Figure 5c) than when using the additive K_2_SO_4_ (Figure 5a). However, the use of both one and the other additive enabled us to reach the degree of hydration 0.99 after 28 days.

The anhydrite binder obtained at the temperature of 800 °C with the use of activators hydrated much faster compared to the anhydrite binder without additives. After 3 days, the degree of hydration increased eight times (Figure 5b,d).

When using the activators K_2_SO_4_ and Na_2_SO_4_, complex salts were formed during hydration: syngenite K_2_Ca(SO_4_)_2_·H_2_O and glauberite Na_2_Ca(SO_4_)_2_. The anhydrite binder hydrated according to the reaction Equations (2)–(5).

(2) $${2K}^{+}+{\mathrm{SO}}_{4}^{2-}+{\mathrm{CaSO}}_{4}+H_{2}O\;\to K_{2}{\mathrm{SO}}_{4}∙{\mathrm{CaSO}}_{4}∙H_{2}O$$

(3) $$K_{2}SO_{4}∙CaSO_{4}∙H_{2}O+H_{2}O\to 2K^{+}+SO_{4}^{2-}+CaSO_{4}∙2H_{2}O$$

(4) $$2Na^{+}+SO_{4}^{2-}+CaSO_{4}+H_{2}O\to Na_{2}SO_{4}∙CaSO_{4}+H_{2}O$$

(5) $$Na_{2}SO_{4}∙CaSO_{4}+H_{2}O\to 2Na^{+}+SO_{4}^{2-}+CaSO_{4}∙2H_{2}O$$

**Figure 6:**
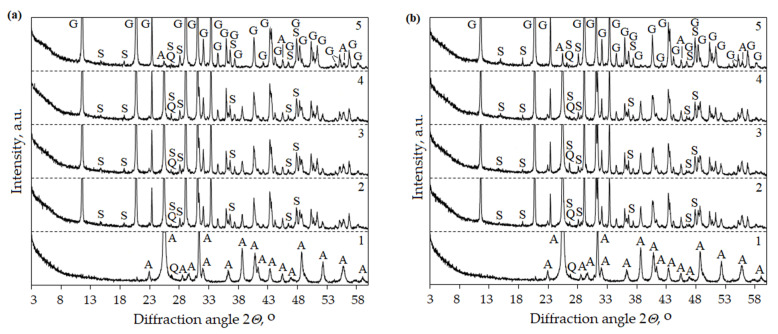

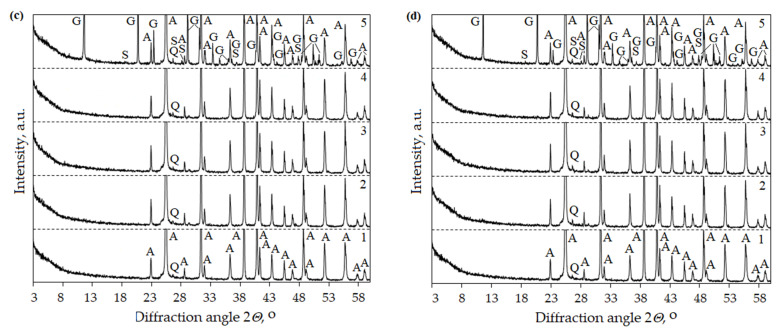
XRD patterns of anhydrite produced at 500 °C using 1% (**a**) and 2% (**b**) K_2_SO_4_ and anhydrite produced at 800 °C using 1% (**c**) and 2% (**d**) K_2_SO_4_. Indexes: A—anhydrite CaSO_4_ (PDF 00-037-1496), Q—quartz SiO_2_ (PDF 00-046-1045), S—syngenite K_2_Ca(SO_4_)_2_·H_2_O (PDF 04-011-9876), G—gypsum (PDF 00-033-0311). 1—anhydrite, 2—anhydrite binder after 10 min hydration, 3—anhydrite binder after 20 min hydration, 4—anhydrite binder after 60 min hydration, 5—anhydrite binder after 1 day hydration.

**Figure 7:**
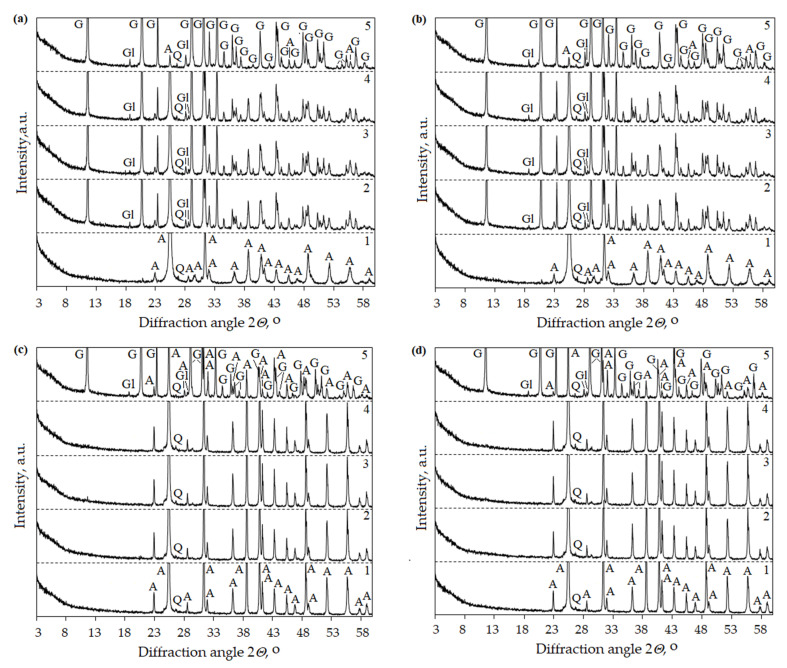
XRD patterns of anhydrite produced at 500 °C using 1% (**a**) and 2% (**b**) Na_2_SO_4_ and anhydrite produced at 800 °C using 1% (**c**) and 2% (**d**) Na_2_SO_4_. Indexes: A—anhydrite CaSO_4_ (PDF 00-037-1496), Q—quartz SiO_2_ (PDF 00-046-1045), Gl—glauberite Na_2_Ca(SO_4_)_2_ (PDF 00-019-1187), G—gypsum (PDF 00-033-0311). 1—anhydrite, 2—anhydrite binder after 10 min hydration, 3—anhydrite binder after 20 min hydration, 4—anhydrite binder after 60 min hydration, 5—anhydrite binder after 1 day hydration.

XRD analysis confirmed the formation of syngenite and glauberite. During the hydration of the anhydrite binder obtained at a temperature of 500 °C and using both 1% and 2% of the activator K_2_SO_4_, syngenite was recorded after 10 min (Figure 6a,b). Meanwhile, during hydration of the obtained anhydrite binder at 800 °C, syngenite was recorded after 1 day. Similar XRD results were obtained using the activator Na_2_SO_4_ when glauberite formed during hydration (Figure 7).

Thus, it is possible to state that the activators K_2_SO_4_ and Na_2_SO_4_ had a large effect on the hydration of the anhydrite binder at its early age (up to 3 days). The presence of the activators of either K_2_SO_4_ or Na_2_SO_4_ almost had no influence on the compressive strength after 28 days.

### 3.3. Full Factorial Design and Statistical Methods

The purpose of this part was to use full factorial design and statistical methods to investigate the impact of the independent factors on the response and develop a mathematical model for predicting response values within the defined limits.

Three independent factors, the calcination temperature of FGD gypsum (*x*_1_), the hydration time (*x*_2_) and the amount of activator (*x*_3_), were chosen. The impact of these factors on the compressive strength of the anhydrite binder using the activators K_2_SO_4_ (*Y*_1_) and Na_2_SO_4_ (*Y*_2_) was investigated using a 2^3^ full factorial design. The used factors and their levels range are shown in Table 3.

The design matrix for three independent factors is presented in Table 4 for the eight experimental runs using the activators K_2_SO_4_ and Na_2_SO_4_.

The regression equation based on the first-order model with three factors and their interaction terms is given with the following expression [43]:

(6) $$Y=b_{0}+b_{1}x_{1}+b_{2}x_{2}+b_{3}x_{3}+b_{12}x_{12}+b_{13}x_{13}{+b}_{23}x_{23}$$

where:

Y—the response: the measured compressive strength of the anhydrite binder using different activators;
*x*_1_, *x*_2_, *x*_3_—the independent factors: calcination temperature, hydration time and amount of activator;
*x*_12_, *x*_13_, *x*_23_—the interaction between independent factors;
*b*_0_—the intercept term;
*b*_1_, *b*_2_, *b*_3_—the linear coefficients for the main factors;
*b*_12_, *b*_13_, *b*_23_—the interaction coefficients for the interaction between corresponding factors.

The regression coefficients *b*_0_–*b*_23_ in Equation (6) were calculated, and the significance of coefficients (Student‘s *t*-test) was determined. All coefficients were significant at a 95% confidence interval.

The regression equations describing the relationship between the significant factors and the compressive strength of the anhydrite binder using the activators K_2_SO_4_ and Na_2_SO_4_ are presented in Equations (7) and (8), respectively:

(7) $$K_{2}{\mathrm{SO}}_{4}\;\;\;\;\;\;\;\;\;{{\hat{Y}}_{1}=11.65+3.16x_{1}+7.32x_{2}+4.63x_{3}+4.96x_{12}-0.71x_{13}+1.66x}_{23}$$

(8) $${\mathrm{Na}}_{2}{\mathrm{SO}}_{4}\;\;\;\;{{\hat{Y}}_{2}=11.16+3.06x_{1}+7.29x_{2}+4.14x_{3}+4.93x_{12}-0.81x_{13}+1.63x}_{23}$$

where:

$\hat{Y}$—the response: the calculated compressive strength of the anhydrite binder, MPa.

The results indicated that the calcination temperature (*x*_1_), the hydration time (*x*_2_), the amount of activator (*x*_3_) and the interaction between calcination temperature and hydration time (*x*_12_) had a significantly strong positive impact on the compressive strength of the anhydrite binder using the activators K_2_SO_4_ and Na_2_SO_4_. The positive impact means that as the mentioned factors and their interaction increase, the compressive strength also increases. The highest value of all coefficients in Equations (7) and (8) had the coefficient for the hydration time (*b*_2_). The value of the mentioned coefficient was +7.32 and +7.29, respectively. This coefficient (*b*_2_) was more than two times than the coefficient *b*_1_ (+3.16 and +3.06, respectively) and more than 1.5 times the coefficients *b*_3_ (+4.63 and +4.14, respectively) and *b*_12_ (+4.96 and +4.93, respectively). Thus, the hydration time (*x*_2_) was the most significant factor compared to the considered factors and their interaction.

The values of the coefficient for the amount of activator (*b*_3_) were also similar in Equations (7) and (8). This means that the effect of the amount of activator on the compressive strength using the considered activators was similar. However, when the activator K_2_SO_4_ was used, the influence of this variable was greater compared to the activator Na_2_SO_4._ This may be influenced by the higher activity of potassium salt [44]. The value of the coefficient for the calcination temperature (*b*_1_) was similar in Equations (7) and (8). This means that the impact of the calcination temperature on the compressive strength using the considered activators was similar, too. The values of the coefficient for interaction between the calcination temperature and hydration time (*b*_12_) were also positive, and the effect on the compressive strength was very strong when the activators K_2_SO_4_ and Na_2_SO_4_ were used.

The coefficient for interaction between the calcination temperature and the amount of activator (*b*_13_) was negative (−0.71 and −0.81). It indicated a negative and weak effect on the compressive strength using the activators K_2_SO_4_ and Na_2_SO_4_.

According to the regression analysis, it can be concluded that the hydration time (*x*_2_) had the strongest influence on the compressive strength using the activators K_2_SO_4_ and Na_2_SO_4._ The interaction between the calcination temperature and hydration time (*x*_12_) and the amount of activator (*x*_3_) had less of an impact on the response, but it was very significant compared to the hydration time (*x*_2_). Meanwhile, when the activator K_2_SO_4_ was used, the amount of activator (*x*_3_) indicated a stronger effect on the compressive strength compared to the activator Na_2_SO_4_. Similar results concerning the use of the activators K_2_SO_4_ and Na_2_SO_4_ were obtained by Leškevičienė et al. [44] and Kamel et al. [45].

The coefficients of determination (*R*^2^) and the mean absolute percentage error (MAPE) between the experimental and calculated values of the compressive strength were used to evaluate the suitability of the obtained models.

The coefficients of determination (*R*^2^) for Equations (7) and (8) were found to be 0.9995 and 0.9996, respectively, and the mean absolute percentage errors (MAPEs) were calculated as 9.53% and 8.48%, respectively. Since the *R*^2^ values were close to 1, and the MAPEs were less than 10%, it is possible to state that the regression Equations (7) and (8) were reliable to forecast the relation between the significant factors, their interaction and the compressive strength of the anhydrite binder.

### 3.4. Model Verification

**Figure 8:**
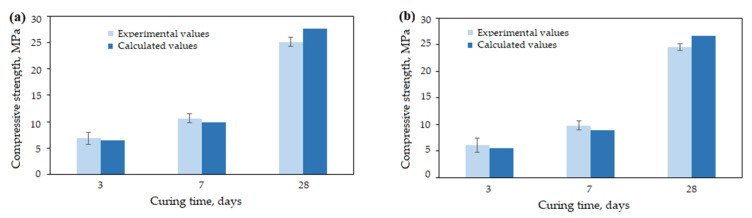
Experimental and calculated values of compressive strength of anhydrite binder using K_2_SO_4_ (**a**) and Na_2_SO_4_ (**b**).

The purpose of this part was to verify the obtained models. The compressive strength was calculated using the obtained Equations (7) and (8) and measured under the same working conditions using the anhydrite binder produced at a temperature of 700 °C and using 2% of activator. The results were obtained after 3, 7 and 28 days. The experimental and calculated values of the compressive strength are presented in Figure 8. The MAPEs of the compressive strength of the anhydrite binder using the activators K_2_SO_4_ and Na_2_SO_4_ were 7.46% and 9.39%, respectively. This shows a good agreement between the experimental data and the calculation using the obtained equations.

In summary, the mathematical model using multiple linear regression could be used to predict the compressive strength of the anhydrite binder within the defined limits.

## 4. Conclusions

The obtained FGD gypsum was of high purity and could be used to produce an anhydrite binder. A 2^3^ full factorial design and statistical methods were used to investigate the impact of the main factors, the calcination temperature of FGD gypsum (500–800 °C), the hydration time (3–28 days) and the amount (0–2%) of activators (K_2_SO_4_ and Na_2_SO_4_), and their interactions on the compressive strength of the anhydrite binder.

The statistical analysis showed that the calcination temperature, hydration time, amount of activator and the interaction between the calcination temperature and the hydration time had a significantly strong positive impact on the compressive strength of the anhydrite binder using the activators K_2_SO_4_ and Na_2_SO_4_. The results indicated that the hydration time had the strongest positive effect on the response. The activator K_2_SO_4_ had a greater influence on the compressive strength than the activator Na_2_SO_4_.

A mathematical model using multiple linear regression was developed. The coefficients of determination (*R*^2^ is close to 1) and the mean absolute percentage error (MAPE is less than 10%) indicated that the obtained model was suitable to predict the compressive strength of the anhydrite binder produced from FGD gypsum within the defined limit values of the considered factors.

## Figures and Tables

**Table 1:** Chemical composition of FGD gypsum.

Constituents, wt. %					
CaO	SO_3_	Al_2_O_3_	Fe_2_O_3_	SiO_2_	Ign. Loss
33.50	46.0	0.31	0.15	1.25	20.14

**Table 2:** Compressive strength of anhydrite binder produced at different calcination temperatures.

Hydration Time, Days	Compression Strength, MPa				
	Calcination Temperature, °C				
	500	600	700	800	900
3 days	2.04	2.00	1.02	0.51	-
28 days	4.10	8.68	10.40	21.27	26.5
28 days (dry samples)	10.81	14.32	17.97	27.63	34.6

**Table 3:** Factors and levels.

Independent Factor	Units	Coding	Range	
			High Level (+1)	Low Level (−1)
Calcination temperature	°C	*x*_1_	800	500
Hydration time	days	*x*_2_	28	3
Amount of activator	%	*x*_3_	2	0

**Table 4:** Design matrix and results of the experiment.

Run	Independent Factor						Compressive Strength, MPa	
	Actual Values			Coded Values				
	Calcination Temperature, °C	Hydration Time, Days	Amount of Activator, %	x_1_	x_2_	x_3_	Y_1_ Using K_2_SO	Y_2_ Using Na_2_SO_4_
1	800	28	2	1	1	1	32.92	31.60
2	500	28	2	−1	1	1	17.60	16.83
3	800	3	2	1	−1	1	4.55	3.48
4	500	3	2	−1	−1	1	10.05	9.27
5	800	28	0	1	1	−1	21.27	21.27
6	500	28	0	−1	1	−1	4.10	4.10
7	800	3	0	1	−1	−1	0.51	0.21
8	500	3	0	−1	−1	−1	2.20	2.20

## Data Availability

Not applicable.
